# Willingness to switch to long-acting regimens among people with HIV: a systematic review and meta-analysis

**DOI:** 10.1097/QAD.0000000000004556

**Published:** 2026-06-05

**Authors:** Kyra F. Mendes de Leon, Roos D. Klungers, Pythia T. Nieuwkerk, Marc Van Der Valk

**Affiliations:** a Division of Infectious Diseases, Amsterdam University Medical Center, University of Amsterdam; b Institute for Immunology and Infectious Diseases; c Amsterdam Public Health Research Institute; d Department of Medical Psychology, Amsterdam University Medical Center; e Stichting HIV Monitoring, Amsterdam, the Netherlands.

**Keywords:** HIV, long-acting regimen, meta-analysis, people-centered care, systematic review, treatment preference

## Abstract

**Objectives:**

Long-acting regimens (LARs) offer promising alternatives for people with HIV, enhancing autonomy and convenience. Understanding preferences for LAR is essential to guide policy and implementation.

**Design:**

This systematic review and meta-analysis examined willingness among adults with HIV to switch from daily oral antiretroviral therapy (ART) to LAR.

**Methods:**

Following PRISMA guidelines, we included studies of adults with HIV using daily oral ART reporting willingness to switch to existing or hypothetical LAR. The primary outcome was the proportion willing to switch. Articles were searched in PubMed and Embase up to October 2024. Risk of bias was assessed using the RoB-PrevMH tool. A random-effects meta-analysis with a generalized linear mixed model synthesized willingness for LAR. Subgroup analyses explored heterogeneity. Alternative LAR were summarized descriptively.

**Results:**

Searches identified 2038 records, of which 22 studies were included. Most studies were conducted in Europe or North America (96%). Willingness to switch to any LAR was 70% [95% confidence interval (95% CI): 59–79; *I*^2^ = 97.9%]. Furthermore, two-monthly intramuscular injections were the most frequently investigated LAR, with a pooled willingness of 60% (95%CI: 42–76; *I*^2^ = 97.5%). No study-level characteristics explained heterogeneity. Limited studies investigated willingness for alternative LAR.

**Conclusion:**

The majority of people with HIV expressed willingness to switch to LAR, with two-monthly intramuscular injections being the most commonly investigated formulation. Substantial heterogeneity persisted, suggesting that LAR preferences are highly context dependent. Evidence on alternative LAR, including tablets, implants and intravenous infusions, is limited. Low and middle-income countries were underrepresented, highlighting the need for geographically diverse research to better understand context-specific preferences and thereby inform treatment policy.

## Introduction

HIV treatment strategies prioritize sustained viral suppression and increasingly emphasize quality of life for people with HIV [1]. Advances in antiretroviral therapy (ART) have substantially improved treatment efficacy and long-term virological control [2]. For the majority of people with HIV, current oral regimens are well tolerated, effective, and have contributed to a life expectancy approaching that of the general population [3]. Nevertheless, suboptimal medication adherence continues to impede sustained viral suppression. In addition, daily ART is associated with stigma, anxiety concerning missed doses, and persistent reminders of one's HIV status [4]. Accordingly, long-acting regimens (LAR) offer a novel treatment modality aimed at overcoming these challenges [2].

In 2021, the HIV treatment paradigm shifted with the approval of Cabotegravir (CAB) and Rilpivirine (RPV), the first injectable LAR [5]. These regimens have shown high acceptability, largely due to the convenience of reduced dosing frequency [6,7] and lower cognitive and emotional demands compared with daily ART [8,9]. However, access to LAR remains unequally distributed. These treatments are largely unavailable in low and middle-income countries (LMICs), even though approximately two-thirds of people with HIV reside there [10]. Acceptability of LAR in LMICs has been reported to be higher compared to high-income countries (HICs), highlighting the urgent need to ensure equitable access [11]. Although voluntary licensing agreements represent an important step toward enabling generic production of LAR in LMICs, persistent barriers particularly pronounced in these settings including cost, cold chain requirements, trained personnel and infrastructure for injectable administration, risk perpetuating the recurrent access delays to novel antiretroviral therapies in these settings [12].

Although CAB and RPV have demonstrated acceptability, a range of other LAR with varying administration schedules and modalities are in development, including injections, implants, infusions, and tablets [13]. These may offer distinct advantages and contribute to a more diverse therapeutic landscape, allowing individuals to choose regimens that best align with their personal needs and circumstances [14].

Offering multiple treatment options enhances the sense of autonomy [15]. According to self-determination theory, motivation increases when autonomy is experienced [16]. Perceived autonomy is associated with medication adherence, mediated by resilience [17,18]. This emphasizes the importance of fostering autonomy within treatment. Incorporating the perspectives of people with HIV into treatment development represents one approach to achieving this. This is in line with the principles of people-centered care, stating that healthcare must be responsive to needs and preferences of people with HIV [19].

Understanding willingness to use both current and future LAR across different settings and populations is therefore critical to ensure that individual preferences are reflected inclusively in novel treatment options and strategies. To date, no systematic review has summarized the available evidence on this topic. Accordingly, we aim to assess willingness among individuals with HIV to switch to LAR through a systematic review and meta-analysis.

## Materials and methods

### Guidelines

This systematic review was conducted in accordance with the guidelines of Preferred Reporting Items for Systematic Reviews and Meta-Analyses (PRISMA) [20].

### Selection criteria

Studies were eligible if they included adults (≥18 years) with HIV who were on daily oral ART and assessed their willingness to switch to LAR. All LAR formulations and dosing schedules were considered, and the primary outcome was defined as the percentage of participants indicating willingness to switch. Studies were required to include at least 95% of participants on daily oral ART to ensure representativeness of the population eligible to switch to LAR. This prevented inclusion of studies in which a substantial proportion of participants were already on LAR or not on daily oral ART, and thus not representative of the intended study population. Both studies that reported willingness to switch in real-world implementation settings and in hypothetical scenarios, including questionnaires and discrete choice experiments (DCEs) were included. For DCE studies, an opt-out option was required. This allowed participants to choose either a LAR alternative or their current daily oral treatment, whereas DCEs without an opt-out limited choices solely to LAR alternatives. Inclusion of the opt-out therefore enabled estimation of the proportion of participants willing or likely to switch to LAR compared with their current daily oral regimen.

Nonpeer-reviewed studies were excluded. No regional restrictions were applied to capture a diverse range of populations.

This systematic review and meta-analysis was based exclusively on previously published literature. All studies included in the review were reported to have obtained appropriate ethical approval and informed consent from participants. Therefore, no additional ethical approval or informed consent was required for this study.

### Search strategy

Relevant articles were identified through systematic searches of PubMed and Embase up to October 21, 2024, and published conference abstracts were identified up to October 28, 2024, with assistance of a clinical librarian. The search string contained several terms, including “HIV,” “long-acting ART,” and “patient preference,” combined using Boolean operators and supplemented by Medical Subject Headings such as “HIV,” “decision making,” and “choice behavior.” Grey literature was not searched due to concerns regarding methodological quality, which may introduce bias and reduce overall robustness of the evidence. The full search string is provided in the supplementary materials. Duplicates were identified with DedupEndNote (Version 1.1.0) [21].

### Selection process

Titles and abstracts retrieved through the search strategy were independently screened by two authors (K.M.dL and R.K.). Articles retrieved from PubMed and Embase were uploaded to Rayyan, a web-based systematic review management tool for screening [22]. In cases of multiple reports from the same authors with overlapping study data, the most recent or comprehensive report was included. Two authors (K.M.dL and R.K.) verified up to October, 2025 whether the abstracts selected had been published as full-text papers. Conflicts between the researchers were resolved through discussion. Studies that met the inclusion criteria, or those for which eligibility was unclear, were further assessed by obtaining the full text.

### Data extraction

Data were extracted using a structured form in Microsoft Excel (version 2408; Microsoft Corporation, USA). Data extraction was independently performed by two authors (K.M.dL and R.K.), and discrepancies were resolved through discussion. The primary outcome, the proportion of participants willing to switch to LAR, was recorded for each study, along with the type of LAR evaluated. Additional study characteristics included title, first author, type of publication (e.g., full-text peer-reviewed articles, abstracts, or conference abstracts), study design (hypothetical, DCE, or implementation), study objectives, inclusion and exclusion criteria, sample size, study location, study period i.e., the timeframe in which the data were collected), and source of funding (industry or nonindustry).

### Quality assessment

Risk of bias (RoB) was assessed using the Risk of Bias in Studies Estimating the Prevalence of Mental Health Disorders (RoB-PrevMH) tool [23]. The RoB-PrevMH was selected, as it was specifically developed for prevalence studies, offering a structured, domain-based assessment that enables systematic classification of studies into low, high, or unclear RoB categories. Unlike tools such as the Joanna Briggs Institute (JBI) checklist [24], which primarily inform inclusion or exclusion decisions, the RoB-PrevMH allows for stratified quality appraisal and examination of its influence on observed estimates. This validated instrument evaluates potential sources of bias across two domains: selection bias and information bias. Selection bias included two items: representativeness of the sample frame and representativeness of the responders, reflecting the degree to which the study population corresponded to the population the study aimed to represent. Information bias included one item concerning the measurement of the condition, which evaluated whether the condition of interest was measured in a valid and reliable manner across all participants. Two authors (K.M.dL and R.K.) independently assigned RoB ratings, and discrepancies were resolved by discussion.

For each study, a combined RoB rating was assigned to facilitate inclusion in the meta-analysis. This combined rating was derived from the three individual RoB assessments. If two out of three items of the RoB assessments had the same judgment (i.e., low, unclear, or high), that judgment was used as the combined RoB. In cases where all three assessments differed (i.e., one low, one unclear, and one high), the overall judgment was classified as unclear.

### Statistical analysis

Study characteristics were summarized descriptively. Only specific LAR options for which data about willingness to switch were provided by data from at least five studies were included in the quantitative analysis, ensuring sufficient power for random-effects meta-analysis [25]. This included both preference for individual LAR formulations and composite estimates in which multiple LAR formulations or dosing frequencies were aggregated into a single overall preference for LAR versus daily oral ART; the latter were collectively referred to as ”any LAR." For LAR options for which preference data were provided by fewer than five studies, findings were summarized descriptively.

A random-effects meta-analysis was conducted using a generalized linear mixed model (GLMM) for single proportions on logit-transformed data, accounting for both within-study variability and between-study heterogeneity. The primary outcome, willingness to switch to LAR, was expressed as a percentage with corresponding 95% confidence intervals (95% CIs) and visually summarized using forest plots. Between-study variance (tau^2^) was estimated using the maximum likelihood method, and heterogeneity was quantified with Higgins and Thompson's *I*^2^ statistic, with values of 25–50, 51–75, and 76–100% interpreted as low, moderate, and high heterogeneity, respectively. Within-study variance was calculated using the Clopper-Pearson method for binomial proportions.

To explore potential sources of heterogeneity, subgroup analyses were conducted. Studies were compared by combined risk-of-bias ratings to examine the influence of study quality. Furthermore, studies were compared by geographic region stratified into the two most represented regions in the literature, the United States (US) and the European Union (EU). Study design was considered, distinguishing between hypothetical scenarios, DCEs, and actual implementation studies. The influence of industry funding was also examined based on funding information provided in each study. For studies investigating preferences for currently available two-monthly injections, a sensitivity analysis was performed restricted to hypothetical preference studies, given that real-world implementation studies may yield systematically different estimates than hypothetical preference studies [26]. Furthermore, for studies exclusively investigating either one-monthly or two-monthly intramuscular injections, willingness was compared across dosing intervals, given that longer dosing intervals may be more preferred [27,28]. Studies investigating two-monthly injections were further categorized by the period of data collection, either before or after 2021, reflecting the introduction of LAR; studies conducted prior to 2021 assessed LAR as a hypothetical future option, whereas studies conducted after 2021 evaluated LAR as a realistic treatment alternative. Acknowledging that implementation varied considerably across countries, studies were additionally stratified by whether two-monthly intramuscular injections were available in the respective country at the time of data collection. Furthermore, since actual uptake of two-monthly injections could only occur after market introduction, inclusion of such studies in timeframe-based subgroup analyses would have confounded implementation period with study design. These analyses were therefore restricted to hypothetical preference studies. If data were missing for a study or information regarding subgroup classification was unavailable, the study was excluded from that specific analysis.

Publication bias was evaluated using funnel plots and Egger's test for asymmetry.

Two-sided *P*-values less than 0.05 were considered statistically significant. Data analyses were conducted using RStudio version 4.4.3 and meta-analyses were performed using the *metaprop* function in the *meta* package.

## Results

**Fig. 1:**
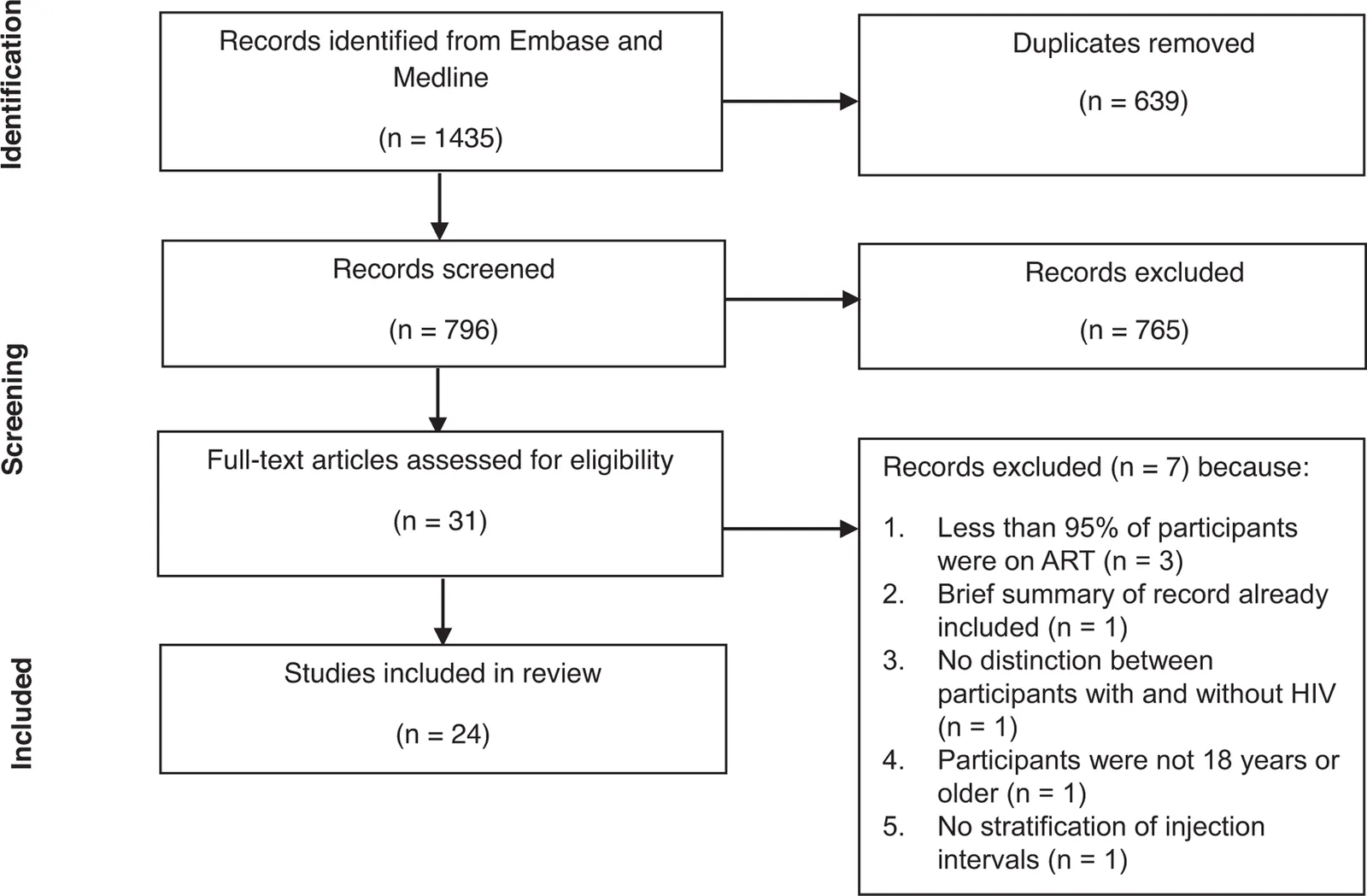
PRISMA (Preferred Reporting Items for Systematic Reviews and Meta-Analyses) diagram.

A total of 2038 records were identified through database searches for study selection. After removing duplicates, 1399 records remained for title and abstract screening. Following this initial screening, 35 full-text articles were assessed for eligibility. Of these, 23 studies met the inclusion criteria and were included in the review. The study selection process is summarized in Fig. 1, which presents the PRISMA (Preferred Reporting Items for Systematic Reviews and Meta-Analyses) flow diagram. During full-text screening, seven articles were excluded. Three did not meet the criterion that at least 95% of participants were on ART, one was a duplicate summary, one did not stratify willingness to switch by HIV status, one included participants less than 18 years of age, and one did not distinguish between dosing intervals.

The majority of included studies were conducted in Europe or North America (96%). One study by Carracedo *et al.*[29] stratified participants by region, including both the United States and Spain, which were retained as separate groups given planned subgroup analyses by region and known differences in healthcare systems between the US and Europe. All studies assessed willingness to switch to intramuscular injectable LAR; only four studies also examined other LAR modalities. In two studies, participants were offered CAB and RPV and made real-life switching decisions, whereas the remaining studies assessed hypothetical willingness to switch. Of the included studies, eight were industry-funded, thirteen were not industry funded, and the funding source was unknown for one study. An overview of study characteristics is provided in Table 1[29–50].

**Table 1:** Study characteristics.

First author	% willingness to switch IM/ two months	% willingness to switch IM/ monthly	% willingness to switch Alternative dosing schedules and modalities: IM, IP, IV, O, or SC	Sample size	Location	Hypothetical (H) or Implementation (I)	Inclusion period
Williams [30]	^a^	^a^	61 (SC weekly); 72 (SC/2 weeks); 84 (SC monthly)	399	USA	H	2011
Dubé [31]	^a^	^a^	86 (IM 1–2 months + SC/6 months)	282	USA	H	2018
Kremer [32]	71	71	^a^	76	The Netherlands	H (DCE)	2020
Akinwunmi [33]	66	^a^		688	Germany; Italy; UK; France	H	2019
Feihel [34]	^a^	31	^a^	102	^a^	I	2021
Soler Carracedo US site [29]	31	31	^a^	55	USA	H	2020-2021
Soler Carracedo Spain site [29]	66	66	^a^	141	Spain	H	2020-2021
Gelhorn [35]	59	59	^a^	554	USA; Canada	H (DCE)	^a^
Matza [36]	61	61	^a^	201	UK	H	2020- 2021
Massaroni [37]	^a^	69	^a^	202	Italy	H	2021
Celesia [38]	^a^	^a^	64 (IM weekly, 1–2 months)	242	Italy	H	2020
Christopoulus [39]	72	^a^	^a^	200	USA	H	2022
Barthold [40]	^a^	^a^	89 (O), (SC), (IM), (IP)	700	USA	H (DCE)	2021-2022
Ishihara [41]	^a^	^a^	40 (SC), (IM), (IP); (O)	667	Japan	H	2021
Mendes de Leon [42]	33	^a^	42 (IM/3 months); 31 (SC/6 months +IM/2 months); 40 (IV/6 months); 44 (IP); 77 (O weekly)	427	The Netherlands	H	2023-2024
Moreno [43]	83	^a^	^a^	271	Spain	H	2022
Cocohoba [44]	59	59	^a^	17	USA	H	^a^
Castelli [45]	24	^a^	^a^	82	Italy	I	2022
Stout [46]	56	56	^a^	155	USA	H	2023
Fisk-Hoffman [47]	^a^	^a^	63 (IM 3 months)	314	USA	H	2020- 2022
Slama [48]	74	^a^	^a^	100	France	H	2018
Sciannameo [49]	^a^	^a^	92 (IM 1–6months)	804	Argentina	H	2021
Collins [50]	63	^a^	^a^	103	USA	H	2022- 2023

IM, intramuscular injections; IP, implants; IV, intravenous infusions; O, oral tablets; SC, subcutaneous injections.

a Data not available or not assessed.

### Risk of bias

**Fig. 2:**
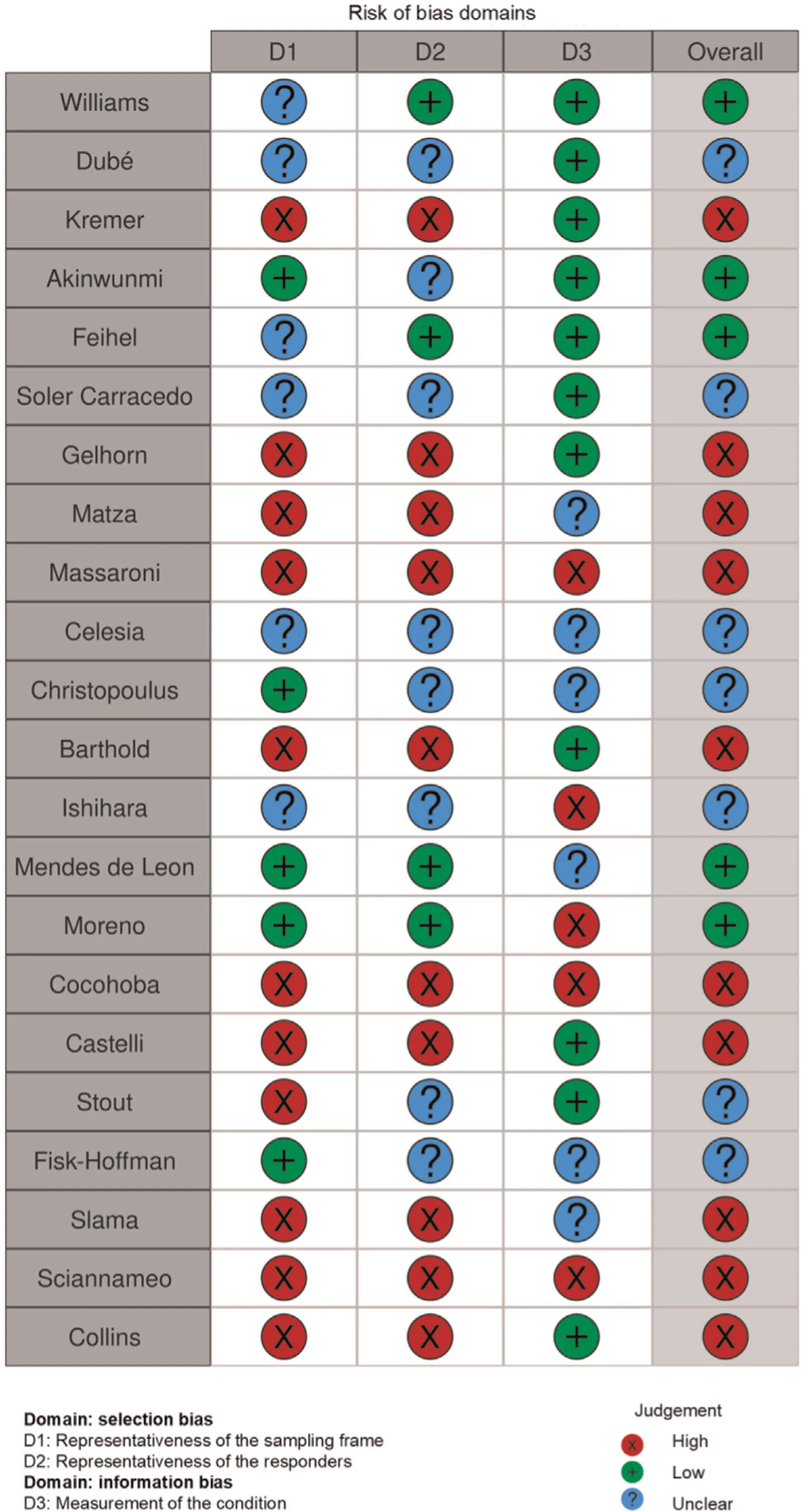
Risk of bias assessment using the RoB-PrevMH tool.

A total of 22 studies were assessed for RoB across three items: representativeness of the sampling frame, representativeness of the responders, and measurement of the condition. One study was rated as low risk across all items, while three were rated as high risk in all items. The remaining 19 studies showed mixed ratings. High RoB was most frequently assigned to the representativeness of the sampling frame and responders (11 and 10 studies, respectively). In contrast, the measurement of the condition was rated as low risk in 11 studies. Unclear RoB was common across all items. RoB was visualized using the robvis tool (Fig. 2) [51].

### Willingness for any long-acting regimens

**Fig. 3:**
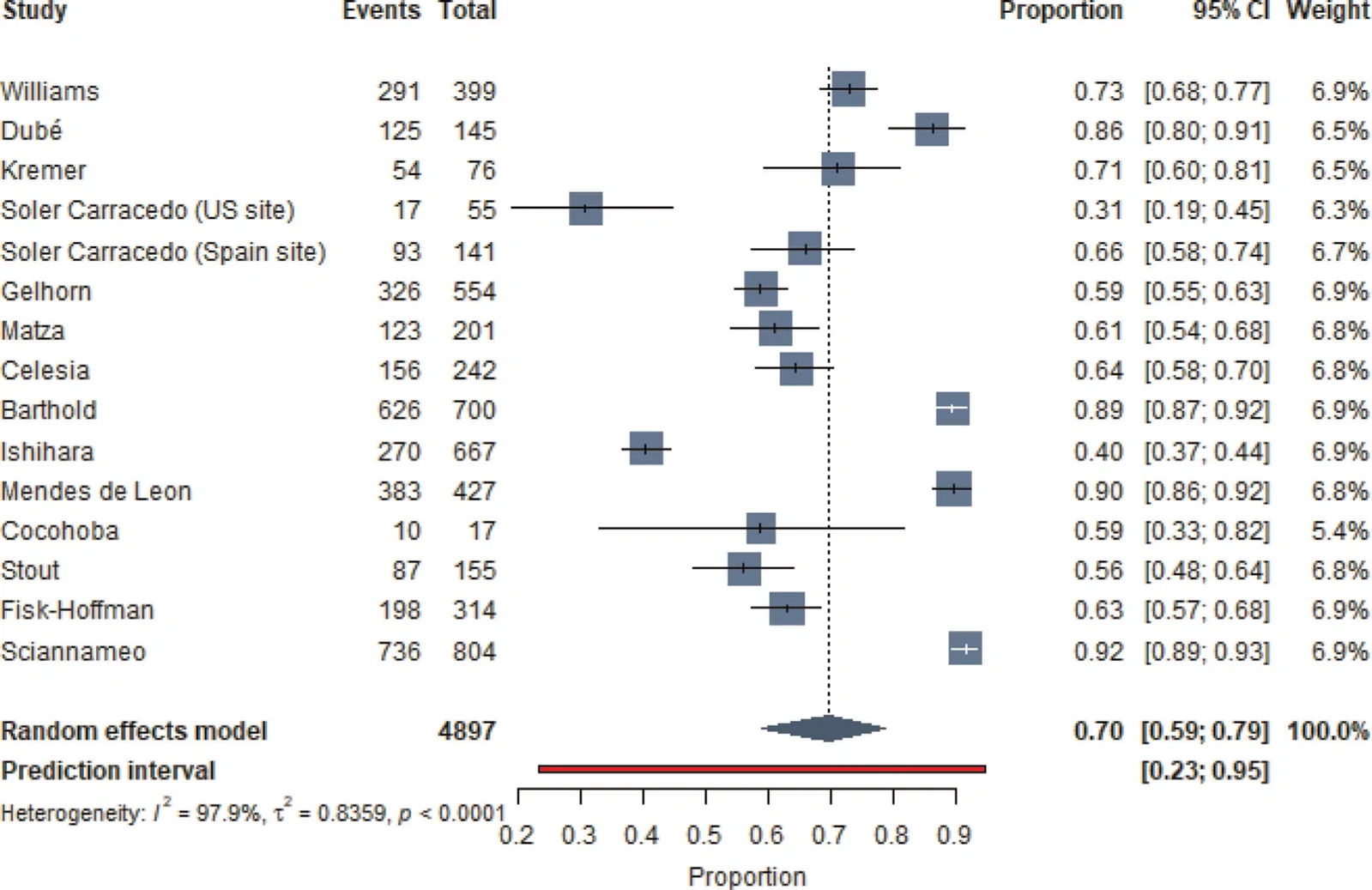
Pooled willingness to switch to any LAR.

Fourteen studies assessed willingness for multiple LAR options or dosing intervals. Eight examined different intramuscular injection intervals and reported a single aggregate willingness estimate across dosing schedules; seven of these compared 1 and 2-monthly dosing and one compared 1 and 6-monthly dosing. The remaining six studies evaluated willingness for intramuscular injections alongside other formulations, including implants, intravenous (i.v.) infusions, oral tablets, and patches, with varying dosing intervals, and reported an aggregate proportion willing to choose any LAR option compared to daily oral therapy. Across these 14 studies, pooled willingness to switch to any LAR option was 73% (95% CI: 61–82; *I*^2^ = 98%) (Fig. 3).

### Willingness for two-monthly injectable intramuscular long-acting regimens

**Fig. 4:**
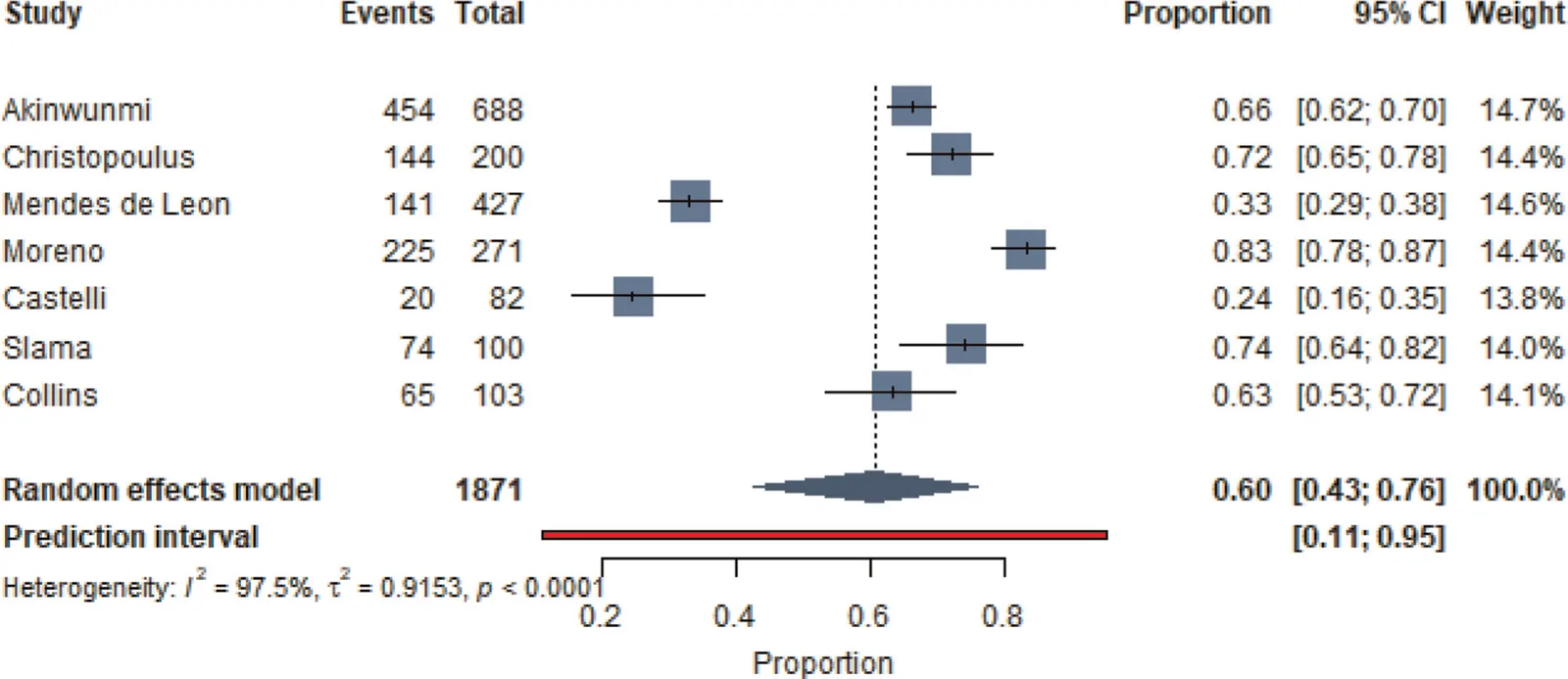
Pooled willingness to switch to two-monthly intramuscular injections.

A total of seven studies, comprising 1871 participants, were included in the meta-analysis evaluating willingness for two-monthly intramuscular injections. The pooled proportion of willingness for two-monthly LAR was 60% (95% CI: 43–76) using a random-effects model. The highest willingness was reported in Moreno *et al.*[43] at 83% (95% CI: 78–87), and the lowest in the study of Castelli *et al.*[45] at 24% (95% CI: 16–35). Between-study heterogeneity was *I*^2^ = 97.5% (Fig. 4).

### Subgroup analyses of willingness for any long-acting regimens

Subgroup analyses according to RoB showed no significant differences in willingness for any LAR between studies with low RoB (83%; 95% CI: 0–100; *k* = 2), studies with high RoB (75%; 95% CI: 54–89; *k* = 6), and studies with unclear RoB (59%; 95% CI: 41–76; *k* = 6) (*P* = 0.123) (Figure S1).

Subgroup analyses comparing studies conducted in the United States showed no significant difference in willingness for LAR compared to studies conducted in Europe (*P* = 0.612). Studies from the United States (*k* = 8) reported a willingness of 67% (95% CI: 49–82), whereas studies from Europe (*k* = 5 reported a willingness of 72% (95% CI: 52–86) (Figure S2).

The majority of included studies assessed willingness to switch to LAR in a hypothetical scenario, yielding an estimate of 68% (95% CI: 54–80; *k* = 11). Studies using DCEs reported a pooled willingness of 76% (95% CI: 24–97; *k* = 3). Differences between hypothetical and DCE-based studies were not statistically significant (*P* = 0.537) (Figure S3).

Industry-funded studies did not differ significantly from nonindustry-funded studies in willingness for LAR (*P* = 0.629). Industry-funded studies (*k* = 7) reported a willingness of 72% (95% CI: 56–84), whereas studies that were not industry-funded (*k* = 6) reported a willingness of 67% (95% CI: 40–86) (Figure S4).

### Subgroup and sensitivity analysis of willingness for intramuscular injections

When the analysis of willingness to switch to currently available two-monthly injections was restricted to hypothetical preference studies, excluding real-world implementation studies, the pooled willingness to switch was 66% (95% CI: 51–79; *I*^2^ = 97.5%) (Figure S5).

When comparing intramuscular injection dosing intervals, no significant difference was found in willingness to receive two-monthly compared with monthly regimens (*P* = 0.643). Willingness for monthly injections was 50% (95% CI: 0–100; *k* = 2), compared with 60% (95% CI: 38–79; *k* = 7) for two-monthly injections (Figure S6).

No significant difference in willingness was observed between studies conducted before and after approval of CAB and RPV in 2021 (*P* = 0.688). Studies conducted before 2021 reported a willingness of 69% (95% CI: 18–96; *k* = 2), compared with 64% (95% CI: 28–89; *k* = 4) in studies conducted in 2021 or later (Figure S7).

Studies were also stratified according to whether LAR had been implemented in each country, acknowledging that implementation did not occur uniformly in 2021 across all settings. Willingness to switch to LAR did not differ significantly between countries where LAR had already been implemented (56%; 95% CI: 13–92; *k* = 3) and those where it had not yet been implemented (75%; 95% CI: 48–91; *k* = 3) (*P* = 0.688) (Figure S8).

There was no evidence of asymmetry in the funnel plot (Figure S9), and the Egger's regression test for funnel plot asymmetry was not statistically significant (*P* = 0.3513). Therefore, publication bias was not suspected in this meta-analysis.

### Willingness for alternative long-acting regimens schedules and modalities

Of the 22 included studies, 21 assessed willingness for intramuscular injections. Of these, 18 studies evaluated willingness for the currently available two-monthly regimen, and 11 studies assessed a monthly dosing schedule; both dosing intervals were included in the meta-analysis. However, some studies examined dosing intervals jointly rather than in isolation, precluding attribution of willingness to a specific interval. Consequently, the number of studies contributing to each dosing-interval-specific meta-analysis was lower than the total number of studies assessing that interval. Furthermore, one study evaluated a three-monthly interval, in which 42% of participants indicated willingness to switch in a study conducted in the Netherlands [42].

Four studies included in the systematic review evaluated alternative LAR. Three studies assessed subcutaneous injections, two evaluated implants, two assessed weekly oral formulations, and one evaluated an i.v. infusion.

Preference for subcutaneous injections was assessed for dosing schedules of weekly, every two weeks, monthly, three-monthly, and six-monthly. For weekly subcutaneous injections, 61% of participants expressed willingness for this dosing schedule, increasing to 72% for injections administered every 2 weeks, and to 84% for monthly injections in a study conducted in the United States [30]. For longer dosing intervals, 31% of participants preferred a combination of subcutaneous 6-month injections with two-monthly intramuscular (i.m.) injections in a study conducted in the Netherlands [42], 38% preferred subcutaneous injections every 3 months [29], and 42% preferred subcutaneous injections every 6 months [31].

Two studies assessed willingness for implants. In one study, implants once per year were preferred by 7% of participants in Japan [41]. In the other study, 44% of participants in the Netherlands favored implants every 6 months [42].

Weekly oral formulations were included in two studies and were chosen by 56% of participants in Japan [41] and by 77% of participants in the Netherlands [42], representing the most frequently chosen option among the alternatives in both studies.

One study also evaluated an i.v. infusion every 6 months, which was preferred by 40% of participants compared to daily oral treatment [42].

## Discussion

To the best of our knowledge, this is the first systematic review and meta-analysis to synthesize willingness to switch from daily oral ART to LAR among people with HIV. Across 14 studies that reported only aggregate willingness across different LAR formulations and/or dosing intervals, 70% of people with HIV indicated that they would be willing to switch to any LAR option. Two-monthly intramuscular injections were the most frequently investigated formulation, with a pooled willingness of 60%. These estimates should nonetheless be interpreted with caution given the substantial and largely unexplained heterogeneity across studies. This may reflect the inherently multifactorial and individualized nature of LAR preferences, which likely vary across populations and settings in ways that aggregate-level analyses cannot fully capture.

Contextual factors may explain variability in willingness to adopt LAR, as prior studies have reported higher willingness among participants from LMICs than those from HICs [11]. This has been linked to higher levels of HIV-related stigma in LMICs, which LAR may help mitigate by enabling more private treatment administration [52]. Due to insufficient number of studies from LMIC, willingness for LAR could not be compared between HIC and LMIC. Only one of the 22 included studies was conducted in a LMIC, illustrating broader inequities in access to LAR, which remain largely unavailable despite evidence of higher willingness to adopt them [12]. These findings are particularly concerning given that two thirds of people with HIV reside in LMICs [53]. In many LMICs, gaps in ART coverage persist and LAR has yet to receive regulatory approval or be widely implemented [54]. Health systems in these settings may consequently prioritize expanding ART access and ensuring treatment continuity. Conducting preference research in these contexts is further challenged by structural barriers including constrained health expenditures, limited research infrastructure, and language and literacy challenges [55]. In our study, the inclusion criterion requiring that at least 95% of participants were receiving daily oral ART may have additionally contributed to the underrepresentation of LMICs, given that ART coverage in these settings remains comparatively lower. Contextual factors, including health system organization and cultural attitudes toward HIV, may further shape willingness, as perspectives on treatment are inherently influenced by the broader social and structural context [11]. Consequently, the external validity of preference estimates derived from HICs cannot be assumed. Investment in geographically diverse preference research is therefore essential to ensure global HIV treatment policies are informed by representative, context-specific evidence.

Several studies have investigated factors influencing LAR preferences, consistently showing greater preference for longer dosing intervals [27,28]. This likely reflects the reduced treatment burden and greater convenience of extended intervals, which may enhance overall well being by allowing individuals to devote less attention to daily HIV management [33,56]. In our study, willingness was higher for two-monthly (60%) compared with monthly (50%) injections, although this difference did not reach statistical significance.

A very limited number of studies have evaluated LAR modalities beyond intramuscular injections. Among studies including implants, preferences varied markedly: only 7% of participants in Japan [41] selected implants compared with 44% in the Netherlands [42]. This difference may reflect familiarity, as implants are widely used for contraception in the Netherlands but not widely used for applications in Japan [57]. This aligns with the familiarity heuristic, whereby familiarity increases its acceptability. A similar pattern was seen for weekly oral tablets, which were the most preferred option in studies that included them, likely due to familiarity with oral therapy [41,42]. Within individual studies, preferences for injections generally increased as dosing frequency decreased [30,42,58]. A reduced treatment burden could make less frequent injections more appealing, even when participants have concerns about the injection route, consistent with studies that examined single treatment attributes [28,35]. However, substantial variation between study sites indicates that contextual factors also influence acceptability. In one study, over one quarter of participants reported previous intravenous drug use, compared with 1.5% in the national population [30,59], which may reduce fear of needles and increase acceptance of injectable treatments. Conversely, other studies identified needle fear as a key barrier to uptake [56,60]. Overall, these findings indicate that both individual experiences and broader contextual norms shape preferences for LAR.

Several limitations should be considered when interpreting these findings. First, while the development pipeline increasingly prioritizes LAR [61], the evidence on preferences for future regimens remains limited. This evidence derives from a small number of studies with hypothetical designs and should therefore be interpreted with caution. Nonetheless, incorporating the perspectives of people with HIV during early design and clinical evaluation is imperative. Given that some people with HIV express hesitation toward current injectable options [62], ensuring acceptability across diverse populations is essential. [61] These considerations underscore the need for further research on willingness to adopt these modalities. Second, only one of the 22 included studies was conducted in LMICs. This disproportionate representation limits generalizability of the findings to these regions and highlights an urgent need for research in these settings. Third, approximately one-third of studies were assessed as a high RoB in at least one domain, suggesting that some prevalence estimates may be less robust and should be interpreted with caution. Fourth, considerable heterogeneity was observed in pooled willingness estimates. Despite systematic exploration of study-level characteristics, heterogeneity remained high, suggesting that methodological factors alone are insufficient to account for the observed variability. Clinical heterogeneity could not be fully explored because the meta-analytic synthesis relied on aggregated study-level data, making the observed associations susceptible to ecological bias [63]. Beyond a methodological limitation, this may itself be substantively meaningful, suggesting that willingness for LAR is a fundamentally individualized outcome that cannot be reduced to a single pooled estimate. Fifth, Egger's test did not indicate funnel plot asymmetry, suggesting no evidence of publication bias. However, this result should be interpreted cautiously, as funnel plot-based methods have well documented limitations in the presence of substantial between-study heterogeneity, which can reduce the statistical power to detect asymmetry and may mask potential bias. Consequently, while no publication bias was detected, this finding does not preclude its presence. Another limitation is the variation in recruitment strategies across studies. Some studies relied on social media and peer-network recruitment, which may have introduced self-selection bias and potentially led to an overrepresentation of individuals with a prior interest in LAR. Most studies, however, recruited participants during routine outpatient clinic visits, where all eligible individuals were approached, thereby mitigating the risk of selection bias. Finally, most studies evaluated hypothetical willingness rather than actual uptake. Hypothetical scenarios are susceptible to optimism bias, whereby participants tend to overestimate their likelihood of adopting a recommended behavior, potentially leading to an overestimation of true willingness to switch to LAR [64]. Only two studies measured actual LAR uptake; although they observed differences, these were not statistically significant, and the limited data make this comparison insufficiently robust. The intention-behavior gap describes how individuals’ stated intentions often fail to translate into action due to logistical, personal, or contextual factors [26]. Therefore, it is essential for future research to examine actual switches, which now remain underrepresented, to better capture real-world behavior.

## Conclusion

Approximately two-thirds of people with HIV on daily oral ART expressed willingness to switch to any LAR, though substantial heterogeneity was observed across studies. Given that LAR preferences are highly individualized and difficult to predict, eligible individuals should be informed about this option where the clinical and contextual situation allows. Treatment decisions should subsequently be reached through shared decision-making. This approach is central to realizing the potential of people-centered care. Despite the growing prioritization of LAR in development, current evidence on preferences for alternative LAR remains limited. The underrepresentation of LMICs underscores the need for equitable research and implementation to ensure access across diverse populations and settings.

## Acknowledgements

The authors thank René Spijker from the medical library of the Amsterdam UMC for providing the search string retrieved from Embase and Medline.

K.M.dL, R.K., P.N., and M.vdV. contributed to the conception of this study. M.vdV. is the study chief investigator. K.M.dL and R.K. collected the data. K.M.dL performed the analyses with oversight by P.N. K.M.dL and R.K. prepared the first draft of the manuscript for publication. K.M.dL, R.K., P.N., and M.vdV. contributed to revising the manuscript and approved the final version to be published.

Authors agree to make data and materials supporting the results or analyses presented in their study available upon reasonable request. It is up to the authors to determine whether a request is reasonable. A protocol was prepared for this review but is not publicly available; the review was not registered.

### Conflicts of interest

M.vdV. has received unrestricted research funding from ViiV, Gilead, and MSD and fees for participation in scientific advisory boards from ViiV, Gilead Sciences, and MSD all paid to his institution. K.M.dL, R.K., and P.N. report no competing interests.

## Supplementary Material

Supplemental Digital Content
