# Supplementary material for: Willingness to switch to long-acting regimens among people with HIV: a systematic review and meta-analysis
Source: AIDS. 2026 Jun 5;40(10):1435–45. doi: 10.1097/QAD.0000000000004556 (PMC13566456; doi:10.1097/QAD.0000000000004556)
Supplement: Supplementary file 9 [file aids-40-1435-s009.docx]

 **Fig. S9. Funnel plot of studies included in this meta-analysis**. Points correspond to individual studies, plotted by observed proportion (x-axis) against standard error (y-axis).
